# Strategies for Optimal Implementation of Simulated Clients for Measuring Quality of Care in Low- and Middle-Income Countries

**DOI:** 10.9745/GHSP-D-16-00266

**Published:** 2017-01-26

**Authors:** Anne Fitzpatrick, Katherine Tumlinson

**Affiliations:** a Department of Economics, University of Massachusetts-Boston, Boston, MA, USA.; b The Office of Population Research, Wallace Hall, Princeton University, Princeton, NJ, USA. Now with the Carolina Population Center, University of North Carolina at Chapel Hill, Chapel Hill, NC, USA.

## Abstract

The use of simulated clients or “mystery clients” is a data collection approach in which a study team member presents at a health care facility or outlet pretending to be a real customer, patient, or client. Following the visit, the shopper records her observations. The use of mystery clients can overcome challenges of obtaining accurate measures of health care quality and improve the validity of quality assessments, particularly in low- and middle-income countries. However, mystery client studies should be carefully designed and monitored to avoid problems inherent to this data collection approach. In this article, we discuss our experiences with the mystery client methodology in studies conducted in public- and private-sector health facilities in Kenya and in private-sector facilities in Uganda. We identify both the benefits and the challenges in using this methodology to guide other researchers interested in using this technique. Recruitment of appropriate mystery clients who accurately represent the facility's clientele, have strong recall of recent events, and are comfortable in their role as undercover data collectors are key to successful implementation of this methodology. Additionally, developing detailed training protocols can help ensure mystery clients behave identically and mimic real patrons accurately while short checklists can help ensure mystery client responses are standardized. Strict confidentiality and protocols to avoid unnecessary exams or procedures should also be stressed during training and monitored carefully throughout the study. Despite these challenges, researchers should consider mystery client designs to measure actual provider behavior and to supplement self-reported provider behavior. Data from mystery client studies can provide critical insight into the quality of service provision unavailable from other data collection methods. The unique information available from the mystery client approach far outweighs the cost.

## Abstract (precis)

When properly implemented, use of simulated clients (“mystery clients”) can provide insight into actual experiences of real clients and evaluate quality of care. Successful implementation calls for recruiting mystery clients who represent the facility's clientele, have strong recall of recent events, and are comfortable being undercover data collectors. Developing training protocols and checklists to standardize mystery client behavior and responses is also key.

## INTRODUCTION

Accurate data collection is essential to evaluating potential problems in service delivery, particularly in low-and middle-income countries. Several methodological tools can be used to gather such information, such as facility-level surveys including provider interviews or the client exit interview. Another tool is the *simulated client* design. In this approach, a study team member or assistant pretends to be a real customer (mystery shopper) or client/patient (standardized patient) who seeks services or care according to standard-ized prearranged scripts. During the health care visit, the provider is unaware that the encounter is for research purposes. Following the session, the undercover data collector—henceforth referred to as the mystery client—then reports her observations to the study team.

In the simulated client approach, a study team member pretends to be a real client (or “mystery client”) who seeks services according to standardized prearranged scripts.

The mystery client methodology has been used successfully in several studies to assess the quality of health care delivery and identify areas for quality improvement.^1^^–^^3^ Mystery clients are considered to be a highly reliable and valid method for assessing provider communication skills and behavior.^4^^,^^5^ Mystery clients have been used extensively in medical education to train and assess the competency of doctors.^6^^,^^7^ This methodology has been used in evaluating provider behavior more generally since 1985,^8^^–^^10^ but is currently used less frequently than other methods such as surveys.

Mystery clients allow insight into actual transactions and experiences of real customers or patients. By relying on data recalled by mystery clients, this method overcomes some key concerns with traditional structured interviews or direct observation methods.^11^ For example, in direct observation, provider behavior is confounded by patient behavior. In contrast, mystery clients are trained to display standardized behavior, isolating how providers respond to observationally similar patrons. Similarly, directly observing providers may result in them changing their behavior toward what they believe will impress or please their observers, in a *Hawthorne effect*. In structured questionnaires of real patrons, there may be a desire for the respondent to be polite or positive toward the provider (*courtesy bias)* or they may simply make mistakes in recalling information (*recall bias)*.^12^^–^^14^ In addition, patients and health care providers may not be eager to participate in surveys—patients may not feel well and providers may be in a rush to deliver medicines to the patient—resulting in *response bias* from refusal rates as high as 38%.^15^ Finally, surveys of providers may instead reflect their best intentions or how they have been trained to deliver services rather than what they actually do in practice, a phenomenon called the “know-do gap.”^16^ However, mystery client studies may present special challenges as compared to survey or interview-based designs—particularly in low- and middle-income countries.

The mystery client method overcomes some key concerns with traditional research methods, including the Hawthorne effect and courtesy, recall, and response biases.

In this article, we discuss our experiences from studies conducted in Kenya and Uganda using mystery client designs and identify lessons learned to inform successful implementation of this approach to assess quality of care. In Kenya, within a sample of 19 urban public and private health facilities, mystery clients were employed to assess the validity of standard data collection tools used to measure the quality of family planning service delivery in large-scale demographic surveys^17^; this study revealed harmful provider practices not detected by the standard tools.^18^ In Uganda, mystery clients purchased anti-malarial medicines at nearly 500 informal private-sector outlets to measure counterfeit drug rates; the study included both urban and rural areas.^15^ We augment our experiences with findings and approaches from recent research from other mystery client studies.

## APPROACH

### Prior to the Visit: Ethical Approval

In general, the ethical issues involved will be study-specific. Using mystery clients may be classified as “deceptive research” depending on the design details, for example, if providers are not informed about the study and do not provide informed consent in advance. Therefore, institutional review boards (IRBs) should be consulted and approve all protocols and activities to ensure ethical concerns are addressed. Study team members must be aware that they are acting as observers and must keep all experiences and data strictly confidential both during and after study completion. Shoppers may sign non-disclosure agreements as a condition of employment to enforce the importance of confidentiality. There may also be special challenges in observing public health facilities due to long lines and possible political sensitivity around the evaluation of government facilities. For example, it may also be necessary to obtain ethical approval from the provincial- and district-level ministries of health. Consulting with these agencies may also help relevant stakeholders feel invested in and appreciate the results of the study. Such buy-in may also help prevent concerns of trespassing on government property.

Institutional review boards should be consulted and approve all mystery client protocols and activities to ensure ethical concerns are addressed.

To avoid discontent among respondents (i.e., health care providers), IRBs may require principal investigators (PIs) to disclose the study design in advance and gain consent of providers or facility supervisors without specifying the date of the visit ahead of time. Alternatively, IRBs may require debriefing providers after the transactions are completed. IRBs may also require that, if debriefed, providers maintain the right to have their data withdrawn because the information was collected by deception. If a sufficient number of providers withdraw as a result of forewarning or debriefing, then the generalizability of results may be in question—particularly if either low- or high-quality providers differentially withdraw.

Institutional review boards may require investigators to disclose the mystery client design in advance to providers or debrief providers afterward.

In Uganda, the IRB agreed to waive both forewarning and debriefing requirements. This waiver was approved because (1) the societal benefits exceeded potential harms to providers due to a dearth of evidence on drug quality, and (2) the deception itself was minor and protocol sufficiently mimicked real-life interactions. Thus, potential harm to subjects was minimal. Signing informed consent before or after the study may also have provided identifying information, risking confidentiality if data were compromised. By contrast, in Kenya, the IRB required informed consent from all facility managers and also required that the identity of individual providers be kept strictly confidential to avoid negative consequences for those providers offering low-quality care.

In addition, while giving providers feedback on results could be beneficial to improving their service delivery, such feedback must be given in a confidential manner that does not in any way jeopardize their job security. In Uganda, it was deemed likely that providers would not believe their data were for research purposes only. Thus, informing providers post-facto that deception had occurred may have aroused more anger, fear, or anxiety from providers than simply not informing them at all. The potential harm from debriefing was exacerbated because providers were primarily informal-sector outlet vendors potentially in violation of legal regulations or engaged in illegal activities.

In the Kenya study, aggregate results for the district were presented to facility managers and local health officials for debriefing and identifying areas for quality improvement. However, the PI presented only data aggregated from all 19 facilities to avoid negative consequences for any particular low-performing providers or facilities.

Finally, PIs may consider whether to obtain informed consent from mystery clients themselves. Informed consent is necessary to publish characteristics of shoppers such as age, gender, or education. Although not required in the Kenya study, this information was requested for the Uganda study at a later date by manuscript reviewers to determine the generalizability of results.

### Prior to the Visit: Training

Recruitment and training of appropriate study team members is essential. Although the PI may pose as the shopper himself or herself,^19^ in our view this is undesirable because the PI may subconsciously change the nature of the interaction in line with preexisting hypotheses. Instead, hiring mystery clients with strong recall ability can help ensure accurate data collection. Recall ability can be assessed during hiring by memory tests. In addition, researchers may wish to consider an applicant's educational qualifications, or language abilities, as a practical matter. In Uganda, for example, it was deemed essential that the PI could directly communicate in English with the study team members. Therefore, all study team members were required to demonstrate English reading and writing ability to facilitate data collection. As a result, the average educational levels of mystery clients exceeded those of the average drug shop clientele, potentially affecting the interpretation of results. In the Kenya study, which took place in central Kisumu, mystery clients had to be fluent in the local language of Dholuo to avoid suspicion on the part of local health care providers; as such, data collectors from Nairobi willing to reside for the duration of the study period in Kisumu were ineligible because they spoke Swahili, not Dholuo. PIs may additionally find it helpful to train and hire several more study team members than needed in the event of attrition or illness; this was necessary in the Uganda study but not in Kenya where data collection activities were completed within 2 weeks.

Hiring mystery clients with strong recall ability, which can be assessed with memory tests, is important to ensuring accurate data collection.

Similarly, accurate data collection also implies that providers are unaware the shopper is also a research team member. For example, if mystery clients are intended to represent a particular income or regional group, their language, dress, and even hairstyle must be standardized to match the target demographic's typical appearance. It also will be important to remove accessories that are correlated with wealth while conducting visits (e.g., wedding rings, watches, and cell phones, among others). This may be challenging; for example, standards of dress may differ between urban and rural areas. In situations where language or dialect varies substantially across areas, researchers may consider using multiple study teams or adjusting protocol to reflect differing norms. In the Uganda study, 2 different teams with different local language abilities were used. If using multiple teams is not possible, then the PIs should alternatively consider restricting to a more homogeneous geographic area. Finally, depending on the script content, shoppers may also need to appear outwardly healthy to ensure that the providers do not get confused about what ailment to treat.

These safeguards appear to have worked in our studies in Kenya and Uganda. In Uganda, during a separate survey conducted at the same outlets that mystery clients visited, providers reported that, on average, they suspected the mystery client only 3% of the time.^8^ Although there may be concerns that shoppers may stand out more in rural areas, this rate did not statistically differ between urban and rural areas. In Kenya, in only 1 visit out of 134 did a mystery client report that the provider questioned her authenticity.

Adequate time for training study team members—including supervisors—is also important to ensure all shoppers follow protocols exactly and consistently. In our studies, mystery clients received extensive training over 3 to 5 days on the study protocol, including the survey instrument, research objectives, outlet or facility locations, transportation logistics, dress code, confidentiality, and other study policies. Training should also emphasize that shoppers should not prompt providers in an effort to help them perform “better.” Training periods may vary depending on the complexities of the intervention. While our studies used 24–40 training hours, one study reported 250 hours of training.^20^

Generally, mystery clients should feel comfortable in their role as an “undercover” data collector; toward this end, extensive time should be set aside during training for role play and practice using the data collection instrument. Role play activities conducted in front of all mystery clients can also help to ensure shopper behavior is standardized. If possible, create time to pilot test the data collection instrument and allow the mystery clients the opportunity to test out their new role in a location away from the study sites. In Uganda, pilot shopping rounds were both supervised and conducted independently, giving confidence to both shoppers and supervisors that shoppers were prepared for full-study data collection.

During this time, the trainer can also make sure that shoppers are interpreting and answering questions in the same way to ensure standardization and inter-rater reliability. In both Kenya and Uganda, a short checklist was used to help the mystery clients recall aspects of their visit and provide a standard-ized evaluation.^21^ Questions should be as objective as possible to prevent speculation.

In mystery client studies in Kenya and Uganda, checklists were used to help the mystery clients recall aspects of their visit and provide standardized evaluations.

Finally, training must emphasize protocols that protect mystery clients from harm. For example, providers can sometimes recommend intramuscular injections even when not clinically indicated. Similarly, project leaders must ensure that less obvious risks, such as taking a temperature with an unsterilized thermometer, also do not occur. As part of their training, mystery clients need to be prepared with various culturally appropriate and plausible strategies and answers to avoid any procedure that could put them at risk. In addition to physical harm, emotional harm and stress can also be minimized by preparing answers for both expected and unexpected questions. In Kenya, all mystery clients were trained to use culturally appropriate excuses to avoid unwanted services such as injections, implantable contraception, or the intrauterine device; excuses included telling the provider they changed their mind or that they first needed to ask their husband, think about it, acquire sufficient funds, or compare with another facility. In Uganda, shoppers were prepared to answer questions ranging from details on the patient's illness to the shopper's personal background. Standardizing shopper answers also ensures that providers view each shopper identically, regardless of what occurs during the transaction. Conducting focus groups with real customers or patients, interviews with local experts, or receiving support from an anthropologist may help PIs anticipate expected questions and ensure realistic responses in line with cultural norms. In the Ugandan study, the exact language, translations, and excuses used were discussed and validated by the mystery clients during training. In extreme circumstances where risks cannot be sufficiently minimized or providers may not accept refusals to treat, the research design should be modified or a different methodology used. For example, in any study ascertaining the quality of service delivery around actual insertion of an intrauterine device or the implantation or injection of a contraceptive method, the mystery client methodology would not be appropriate.

### Planning the Visit: Sampling Frame

Building a sampling frame can also be particularly challenging in low- and middle-income countries. Many health care outlets lack outward signage; official records may be incomplete; administrative boundaries uncertain. Developing a standardized protocol for building the sample frame of eligible outlets and creating the study plan is important to reduce bias and minimize unexpected events during fieldwork. If data are to be collected more than once from the same location, as is common in audit study designs, study team members may create maps of all study areas, along with a physical description of the outlets or buildings, to help them find the same outlets again. We followed this approach in Uganda. If ethical approvals allow, GPS coordinates can facilitate this process. In Uganda, we validated mapped location information by consulting with local key informants, such as village chiefs or motorcycle taxi drivers, to cross-reference geographic details. The location of health care facilities selected for the Kenya study were widely known to both the local population and the data collectors, who also resided in the area.

### Planning the Visit: Pre-Study Visits

Even if a reliable sample frame already exists, visits to study sites prior to actual data collection are essential for planning how clients approach a provider. Such visits can be conducted in advance by the PI when obtaining informed consent from the facility manager; for studies in which informed consent has been waived, a member of the study team can assess the location in advance of study implementation. Pre-study visits also establish a specific location to conduct the post-visit shopper debriefing with the supervisor, which ideally would occur immediately after each encounter to ensure accurate recall. Establishing a meeting point location also gives shoppers confidence that their supervisors are close by if any complications arise. If debriefing is done orally or with qualitative methods—which can capture additional interesting and important details of the transaction—then identifying quiet areas where confidential discussions can occur is of additional importance.

### During the Visit: Standardized Shopper Behavior

During the visit, all shopper behavior—from words spoken to shopper demeanor—should be completely standardized to be able to make comparisons across providers or visits. Ensuring data are collected according to guidelines over time requires ongoing effort and close monitoring. In both Kenya and Uganda, we reviewed protocol and common responses to questions during daily team meetings to ensure consistency over time and allow for a discussion of how shoppers were feeling. In our studies, supervisors conducted audits and additional checks to ensure that the correct facilities were visited, while being careful not to compromise the mystery client's covert status.

### During the Visit: Collecting Data on Prices

Prices for services or products in developing countries are often not posted and receipts are uncommon. If the research design requires shoppers to make financial payments, then developing strategies to ensure shoppers honestly and correctly report prices is of utmost importance. One option is to trust study team members to honestly report the prices they paid and allow them to bargain for the best price in an effort to balance minimizing study costs with gaining insight to true client experiences. While common in studies of bargaining, this type of protocol could be expensive and may still lead to incorrect data if shoppers switch to cheaper treatments (without notifying the study team). Our approach in both Kenya and Uganda was to instead have multiple shoppers visit the same outlet or facility; this created the impression that the study team could cross-reference prices between shoppers, even though in Uganda, measuring price differences between shoppers at the same provider was the outcome of interest. Researchers considering this approach should ideally have visits conducted by different shoppers several hours (or even days) apart. Multiple visits to the same provider, facility, or outlet are typically feasible but may be difficult if the provider is frequently absent from work or if the facility or outlet has limited operating hours. On occasion, official hours of operation may differ from actual hours.

Similarly, clear protocols and rules need to be established if shoppers are to conduct financial transactions. In Uganda, we set a budget of 10,000 UGX (Ugandan shillings) per transaction. The bills used were in small denominations of used-looking money to minimize the likelihood that providers lacked adequate change to complete a transaction and ensure that the shopper remained inconspicuous. The money was placed in an envelope with the shop and transaction ID labeled on the outside. After the transaction, the shoppers returned the balance to the envelope and accounts reconciled with the price paid. If shoppers required additional funds for a purchase, they were required to return to supervisors. Shoppers were not allowed to use their own money under any circumstances. Separate funds and budgets were used for transportation expenditures. Similar procedures were observed in Kenya.

Clear protocols and rules need to be established if mystery clients are to conduct financial transactions.

In addition, collecting data from real customers, or drug pricing data from vendors, may provide better data and allow for cross-referencing of price data. Additional data collection can also validate assumptions of the experimental design. In the Ugandan context, collecting additional data from real customers validated the assertion that mystery clients behaved similarly to real customers. Another mystery client study examined whether providers altered drug prices or quality if they perceived customers to be of different income levels. To validate provider impressions of shopper income levels, the researchers took daily photographs of shopper outfits that were then evaluated by real customers to test whether mystery clients' appearance reasonably represented socioeconomic status.^22^

### Changing Study Protocols and Rules

Developing good communication is important to ensuring study team members perform as directed. However, listening to concerns of shoppers implies possibly adapting protocol and rules accordingly. For example, during the pilot for the Uganda study, shoppers reported difficulty in understanding the debriefing instrument. As a result, prior to the full study, the debriefing checklist was shortened from 46 items to 18 items and reorganized. In another example, in Uganda it became clear that the bargaining protocol was difficult to enforce; shoppers were worried they were not getting a “good price” because the protocol limited them to 3 rounds of bargaining. Thus, the protocol was simplified and shoppers were allowed to act as they normally would in a bargaining situation with unlimited rounds of bargaining. In Kenya, mystery clients sometimes needed an additional day to collect data on providers with high rates of absenteeism. Other examples of modifications include altering the dress code in urban areas to reflect shopper concerns that their appearance did not reflect local norms.

## CONCLUSION

In summary, the use of mystery clients offers a unique and critical opportunity to accurately meas-ure the quality of service provision. When selecting mystery clients, it is important to choose people who will present realistically and have excellent recall. In addition, careful attention should be paid to strategies that allow mystery clients to feel comfortable in their role and to prevent unwanted exams or procedures. Researchers should appreciate details of data collection and listen to concerns of their team members. Despite these challenges, the unique information available from this type of methodology far outweighs the cost.

## Notes

**Peer Reviewed**

**First Published Online:** 2017 Jan 26
